# Machine and Deep Learning on Radiomic Features from Contrast-Enhanced Mammography and Dynamic Contrast-Enhanced Magnetic Resonance Imaging for Breast Cancer Characterization

**DOI:** 10.3390/bioengineering12090952

**Published:** 2025-09-02

**Authors:** Roberta Fusco, Vincenza Granata, Teresa Petrosino, Paolo Vallone, Maria Assunta Daniela Iasevoli, Mauro Mattace Raso, Sergio Venanzio Setola, Davide Pupo, Gerardo Ferrara, Annarita Fanizzi, Raffaella Massafra, Miria Lafranceschina, Daniele La Forgia, Laura Greco, Francesca Romana Ferranti, Valeria De Soccio, Antonello Vidiri, Francesca Botta, Valeria Dominelli, Enrico Cassano, Charlotte Marguerite Lucille Trombadori, Paolo Belli, Giovanna Trecate, Chiara Tenconi, Maria Carmen De Santis, Luca Boldrini, Antonella Petrillo

**Affiliations:** 1 Radiology Division, Istituto Nazionale Tumori-IRCCS-Fondazione G. Pascale, 80131 Naples, Italy; r.fusco@istitutotumori.na.it (R.F.); t.petrosino@istitutotumori.na.it (T.P.); p.vallone@istitutotumori.na.it (P.V.); m.iasevoli@istitutotumori.na.it (M.A.D.I.); m.mattaceraso@istitutotumori.na.it (M.M.R.); s.setola@istitutotumori.na.it (S.V.S.); davide.pupo@istitutotumori.na.it (D.P.); a.petrillo@istitutotumori.na.it (A.P.); 2 Pathology Division, Istituto Nazionale Tumori-IRCCS-Fondazione G. Pascale, 80131 Naples, Italy; gerardo.ferrara@istitutotumori.na.it; 3 Direzione Scientifica, IRCCS Istituto Tumori Giovanni Paolo II, Via Orazio Flacco 65, 70124 Bari, Italy; annarita.fanizzi.af@gmail.com; 4 SSD Fisica Sanitaria, IRCCS Istituto Tumori Giovanni Paolo II, Via Orazio Flacco 65, 70124 Bari, Italy; 5 Struttura Semplice Dipartimentale di Radiodiagnostica Senologica, IRCCS Istituto Tumori Giovanni Paolo II, Via Orazio Flacco 65, 70124 Bari, Italy; m.lafranceschina@oncologico.bari.it (M.L.); d.laforgia@oncologico.bari.it (D.L.F.); 6 Radiology and Diagnostic Imaging, Istituto di Ricovero E Cura a Carattere Scientifico (IRCCS) Regina Elena National Cancer Institute, 00144 Rome, Italy; laura.greco@ifo.it (L.G.); francescaromana.ferranti@ifo.it (F.R.F.); valeria.desoccio@ifo.it (V.D.S.); antonello.vidiri@ifo.it (A.V.); 7 Breast Imaging Division, IEO Istituto Europeo di Oncologia, 20141 Milan, Italy; francesca.botta@ieo.it (F.B.); valeria.dominelli@ieo.it (V.D.); enrico.cassano@ieo.it (E.C.); 8 Dipartimento di Diagnostica per Immagini, Radioterapia Oncologica ed Ematologia, Fondazione Policlinico Universitario A. Gemelli IRCCS, 00168 Rome, Italy; charlottemargueritelucille.trombadori@guest.policlinicogemelli.it (C.M.L.T.); paolo.belli@policlinicogemelli.it (P.B.); luca.boldrini@policlinicogemelli.it (L.B.); 9 Department of Radiodiagnostic and Magnetic Resonance, Fondazione IRCCS Istituto Nazionale dei Tumori, 20133 Milan, Italy; giovanna.trecate@istitutotumori.mi.it; 10 Department of Medical Physics, Fondazione IRCCS Istituto Nazionale dei Tumori, 20133 Milan, Italy; chiara.tenconi@istitutotumori.mi.it; 11 De Santis Radiation Oncology, Fondazione IRCCS Istituto Nazionale dei Tumori, 20133 Milan, Italy; mariacarmen.desantis@istitutotumori.mi.it

**Keywords:** radiomics, DCE-MRI, CEM, machine learning, deep learning, breast cancer

## Abstract

Objective: The aim of this study was to evaluate the accuracy of machine and deep learning approaches on radiomics features obtained by Dynamic Contrast Enhanced Magnetic Resonance Imaging (DCE-MRI) and contrast enhanced mammography (CEM) in the characterization of breast cancer and in the prediction of the tumor molecular profile. Methods: A total of 153 patients with malignant and benign lesions were analyzed and underwent MRI examinations. Considering the histological findings as the ground truth, three different types of findings were used in the analysis: (1) benign versus malignant lesions; (2) G1 + G2 vs. G3 classification; (3) the presence of human epidermal growth factor receptor 2 (HER2+ vs. HER2−). Radiomic features (n = 851) were extracted from manually segmented regions of interest using the PyRadiomics platform, following IBSI-compliant protocols. Highly correlated features were excluded, and the remaining features were standardized using z-score normalization. A feature selection process based on Elastic Net regularization (α = 0.5) was used to reduce dimensionality. Synthetic balancing of the training data was applied using the ROSE method to address class imbalance. Model performance was evaluated using repeated 10-fold cross-validation and AUC-based metrics. Results: Among the 153 patients enrolled in the studies, 113 were malignant lesions. Among the 113 malignant lesions, 32 had high grading (G3) and 66 had the HER2+ receptor. Radiomic features derived from both CEM and DCE-MRI showed strong discriminative performance for malignancy detection, with several features achieving AUCs above 0.80. Gradient Boosting Machine (GBM) achieved the highest accuracy (0.911) and AUC (0.907) in differentiating benign from malignant lesions. For tumor grading, the neural network model attained the best accuracy (0.848), while LASSO yielded the highest sensitivity (0.667) for detecting high-grade tumors. In predicting HER2+ status, the neural network also performed best (AUC = 0.669), with a sensitivity of 0.842. Conclusions: Radiomics-based machine learning models applied to multiparametric CEM and DCE-MRI images offer promising, non-invasive tools for breast cancer characterization. The models effectively distinguished benign from malignant lesions and showed potential in predicting histological grade and HER2 status. These results demonstrate that radiomic features extracted from CEM and DCE-MRI, when analyzed through machine and deep learning models, can support accurate breast cancer characterization. Such models may assist clinicians in early diagnosis, histological grading, and biomarker assessment, potentially enhancing personalized treatment planning and non-invasive decision-making in routine practice.

## 1. Introduction

Breast cancer remains the most common malignancy among women worldwide, and early detection remains a critical determinant of prognosis. Screening mammography is widely used and has proven effective, but it has notable limitations, particularly in women with dense breast tissue, where lesion detection becomes more challenging due to reduced contrast between tumor and glandular tissue [1,2]. As a result, supplementary imaging techniques such as contrast-enhanced mammography (CEM) and dynamic contrast-enhanced magnetic resonance imaging (DCE-MRI) have gained increasing relevance in clinical workflows [3,4].

CEM enhances lesion conspicuity by combining high-resolution digital mammography with functional information obtained after the administration of iodinated contrast agents. This allows for improved visualization of tumor-related neovascularization and has been shown to increase sensitivity in various clinical scenarios, including dense breasts and diagnostic problem-solving [5,6,7,8,9,10,11,12,13]. DCE-MRI, on the other hand, provides high-resolution, multiparametric information about vascular kinetics, morphology, and tissue heterogeneity, and is considered the most sensitive technique for breast cancer detection. Nevertheless, both techniques come with limitations related to cost, availability, and the need for expert interpretation [13].

Despite advances in imaging, there remains a need for tools that can support objective and reproducible analysis, particularly in complex diagnostic settings involving ambiguous lesions or subtle morphologic features. In this context, radiomics and artificial intelligence (AI) offer new opportunities to extract, quantify, and interpret imaging biomarkers that may not be visible to the human eye. Radiomics enables the extraction of hundreds of quantitative features related to texture, shape, and intensity from standard imaging datasets. These features, when combined with machine learning (ML) or deep learning (DL) algorithms, can be used to predict clinical outcomes, classify lesions, or estimate molecular profiles in a non-invasive and reproducible manner [14,15,16,17,18,19,20,21,22,23,24,25,26].

A growing body of literature has demonstrated the potential of radiomics-based models for breast cancer classification, grading, and molecular subtyping. For instance, studies have shown that radiomic features from MRI or CEM can help differentiate HER2-positive and triple-negative cancers [27], predict hormone receptor status [28], or distinguish benign from malignant lesions with high accuracy [29,30]. However, many of these studies are limited by small sample sizes, the use of a single imaging modality, or the focus on binary classification. Moreover, few studies have systematically compared the performance of different AI models (e.g., ensemble vs. neural networks) across multiple clinically relevant endpoints using radiomic data from both CEM and DCE-MRI.

In this study, we propose a comprehensive radiomics framework that integrates features from both contrast-enhanced mammography and dynamic contrast-enhanced MRI to support breast cancer characterization. Specifically, we evaluated the ability of different machine and deep learning models to predict (1) benign vs. malignant lesions; (2) low-grade (G1 + G2) vs. high-grade (G3) tumors; and (3) HER2-positive vs. HER2-negative cancers.

To ensure robust analysis, we employed standardized radiomic feature extraction using the PyRadiomics platform, dimensionality reduction via Elastic Net regularization, and synthetic balancing using the ROSE technique. Multiple models—including logistic regression, random forest, gradient boosting, neural networks, and decision trees—were trained and validated using repeated cross-validation. By comparing simple interpretable models with more complex architectures, this study also explores the trade-off between accuracy and explainability in radiomics-based breast cancer modeling.

Our findings aim to provide insight into the feasibility, accuracy, and clinical relevance of combining CEM- and MRI-derived radiomic signatures. In this way, we address current gaps in the literature and move toward the goal of developing non-invasive, AI-assisted tools for personalized breast cancer diagnosis and management.

## 2. Methods

### 2.1. Patient Selection

Patient enrollment took place between October 2017 and January 2023. The retrospective, multicenter study was conducted following approval from the local Institutional Review Board (deliberation no. 868, dated 3 September 2020). Participants provided written informed consent.

A total of 154 patients were included in the analysis, each having undergone both DCE-MRI and CEM imaging. The cohort had a mean age of 52.8 years (±12.3), with ages ranging from 25 to 92 years.

The inclusion criteria comprised patients with histologically confirmed breast lesions who underwent both DCE-MRI and CEM imaging as part of their preoperative staging. The study involved several institutions: the Istituto Nazionale Tumori—IRCCS—Fondazione G. Pascale (Naples), the Oncological Institute of Bari (Bari), the Agostino Gemelli University Hospital (Rome), the IFO—Regina Elena National Cancer Institute (Rome), the National Cancer Institute of Milan (Milan), and the IEO—European Institute of Oncology (Milan).

Exclusion criteria included the presence of breast implants, non-removable nipple piercings, pacemakers, clips or other metallic implants, pregnancy or suspected pregnancy, inability to remain still during imaging, history of metal allergy, renal impairment, or ongoing chemotherapy at the time of examination. Furthermore, patients with severe claustrophobia or extreme obesity were considered ineligible for MRI acquisition [26].

### 2.2. Imaging Protocol

The same acquisition protocol was implemented by all centers.

Magnetic resonance images with high spatial and temporal resolution were acquired using a 1.5 Tesla MR scanner equipped with a 16-channel breast coil. The imaging protocol included T2-weighted turbo spin echo sequences (STIR TSE) and multiple T1-weighted gradient echo sequences acquired both before and after intravenous administration of a paramagnetic contrast agent at a dose of 0.1 mmol/kg of body weight. The contrast agent was injected at a rate of 2 mL/s, followed by a 40 mL saline flush at the same flow rate, both delivered via an automated injector.

The breast MRI protocol incorporated at least five post-contrast T1-weighted image acquisitions, performed approximately at 60 ± 10, 120 ± 10, 180 ± 10, 240 ± 10, and 360 ± 10 s following contrast administration [11,13,21,22,26]. Three subtracted image phases were evaluated: early-phase subtraction images acquired at approximately 60 ± 10 s, arterial-phase subtraction images acquired between 120 ± 10 and 180 ± 10 s and delayed-phase subtraction images acquired at around 360 ± 10 s post-contrast injection [11,13,21,22,26]. Details of MR sequence parameters used in this analysis were reported in Table 1.

The CEM protocol included image acquisition in both cranio-caudal (CC) and mediolateral oblique (MLO) projections, initiated two minutes following intravenous injection of an iodinated contrast agent (Visipaque 320; GE Healthcare, Inc., Princeton, NJ, USA) at a dosage of 1.5 mL/kg body weight and an injection rate of 2–3 mL/s. Additional image series were acquired at approximately four and eight minutes post-injection, again in both CC and MLO views.

Each CEM exam comprised a dual-energy acquisition: a low-energy (LE) exposure (26–30 kVp) and a high-energy (HE) exposure (45–49 kVp). The resulting LE and HE images were digitally subtracted to generate recombined images that emphasize areas of contrast uptake, providing functional insight into lesion vascularization.

Post-processing of all CEM images was centralized and conducted at a single institution, encompassing both segmentation and radiomic feature extraction. Radiomic analysis was performed on the CC and the initial MLO view acquired four minutes after contrast administration, a time point corresponding to the optimal visualization of contrast enhancement during the wash-in phase.

### 2.3. Imaging Processing and Radiomic Analysis

For both CEM and DCE-MRI, manual segmentation was performed by two radiologists with extensive experience in breast imaging (ranging from 20 to 25 years). The process was conducted using the 3D Slicer software (version 5.6.1; available at https://download.slicer.org/ accessed on 1 January 2021). Initially, each radiologist carried out the segmentation independently; subsequently, a consensus review was conducted to ensure agreement on the slice-by-slice delineation of the lesions across all imaging planes.

The resulting segmentation masks were employed to define the volumes of interest (VOIs) for the CC and MLO projections in CEM, as well as for each sequence in the DCE-MRI dataset. Examples of segmentation procedures and protocols have been previously documented in earlier studies [21,22].

A total of 851 radiomic features were extracted for each volume of interest, calculated as median values. Feature extraction was performed using the PyRadiomics library integrated within the 3D Slicer image computing platform [14]. Radiomic features were extracted in compliance with the standards proposed by the Imaging Biomarker Standardization Initiative (IBSI), ensuring methodological consistency. The extraction was conducted using a software package that enables the computation of a wide range of features, including first-order statistics, two- and three-dimensional shape descriptors, and various texture metrics. Specifically, texture analysis included features derived from the Gray Level Co-occurrence Matrix (GLCM), Gray Level Run Length Matrix (GLRLM), Gray Level Size Zone Matrix (GLSZM), Gray Level Dependence Matrix (GLDM), and the Neighboring Gray Tone Difference Matrix (NGTDM). Additionally, the software supports wavelet-based filtering to enhance feature representation. Comprehensive information on feature extraction methods and definitions can be found in [14].

The Checklist for EvaluAtion of Radiomics research (CLEAR) was adopted as a structured, step-by-step reporting guideline to ensure transparency, reproducibility, and methodological rigor throughout the radiomics analysis process [31].

### 2.4. Histopathological Analysis

Histopathological evaluation of tissue specimens was considered the diagnostic gold standard. Both tumor grade and HER2 status were assessed through immunohistochemical analysis. Tumor grading was performed according to the modified Scarff-Bloom-Richardson system, as refined by Elston and Ellis, which classifies tumors into three grades based on tubule formation, nuclear pleomorphism, and mitotic count.

HER2 (human epidermal growth factor receptor 2) expression was evaluated using IHC staining, with scores ranging from 0 to 3+. Tumors were classified as HER2-positive when strong complete membrane staining was observed in more than 10% of tumor cells (score 3+). In cases with an equivocal IHC result (score 2+), HER2 amplification was confirmed using fluorescence in situ hybridization (FISH), in accordance with ASCO/CAP guidelines. The identification of HER2 overexpression is clinically relevant, as it is associated with a more aggressive tumor phenotype and potential eligibility for targeted therapies such as trastuzumab.

### 2.5. Statistical Analysis

Three classification outcomes were considered in the analysis: (1) differentiation between benign and malignant lesions; (2) discrimination between low/intermediate-grade tumors (G1 + G2) and high-grade tumors (G3), based on histological grading; and (3) identification of HER2-positive versus HER2-negative breast cancers.

Prior to modeling, features exhibiting high multicollinearity (Pearson correlation coefficient > 0.9) were identified and removed. The remaining features were normalized using z-score standardization to ensure comparability across different scales.

Three binary classification tasks were independently analyzed. Each dataset had a corresponding binary outcome variable, which was used as the dependent variable for model training.

To address dimensionality reduction and identify the most relevant radiomic features, a logistic regression model with Elastic Net regularization was employed. This technique combines the penalties of Least Absolute Shrinkage and Selection Operator (LASSO) and Ridge Regression, enabling both variable selection and coefficient shrinkage. The optimal value of the regularization parameter (lambda) was determined through cross-validation. Features associated with non-zero coefficients at the minimum value of lambda were retained for subsequent modeling [32].

Due to class imbalance in the outcome variables, data balancing was performed using the ROSE (Random Over-Sampling Examples) technique from the ROSE package. ROSE generates synthetic samples for the minority class by sampling from a smoothed bootstrap distribution, thus enhancing model learning and mitigating bias toward the majority class. Balancing was applied exclusively to the training dataset after a 70/30 stratified train-test split.

While data augmentation and image-domain filtering are common in deep learning pipelines, in this study, we focused on handcrafted radiomic features extracted from pre-segmented images.

Therefore, the Radiomic features processing includes:

- Z-score normalization applied to all features to ensure comparability across features and patients.
- Removal of highly correlated features to reduce redundancy.
- Synthetic balancing using the ROSE method to address class imbalance.

Then, nonparametric Wilcoxon–Mann–Whitney test was utilized. Moreover, receiver operating characteristics (ROC) analysis with the calculation of area under the ROC curve (AUC), sensitivity (SENS), specificity (SPEC), positive predictive value (PPV), negative predictive value were calculated (NPV) and accuracy (ACC) was assessed for each feature and for each model.

A total of six machine learning models were trained and evaluated:

- Elastic Net Regularized Logistic Regression (LASSO)
- Random Forest (RF)
- Gradient Boosting Machine (GBM)
- Neural Network (NN)
- Classification and Regression Tree (CART)

This selection provides a balanced comparison of model types under similar training conditions, without introducing excessive architectural complexity for the dataset size.

Each model was trained using the features selected by Elastic Net. Hyperparameter tuning was conducted applying repeated 10-fold cross-validation to ensure robust estimation of model performance (Table 2). The area under the receiver operating characteristic curve was used as the primary performance metric. Class probabilities were calculated for ROC analysis, and optimal classification thresholds were derived using Youden’s index.

The choice of models was guided by:

- Their established effectiveness in radiomics literature for tabular feature classification.
- The need to compare interpretable models (e.g., LASSO, CART) with higher-capacity learners (e.g., GBM, NN).
- Use of grid search and cross-validation to optimize model performance based on AUC or Accuracy metrics.

**Figure 1:**
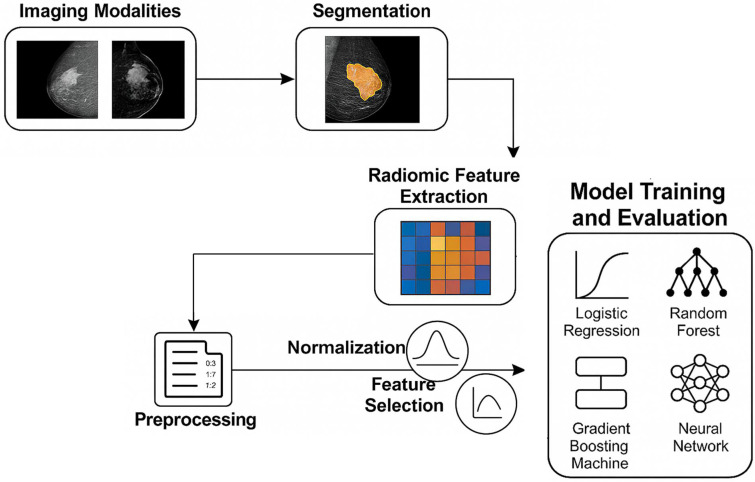
A schematic workflow diagram.

Figure 1 illustrates workflow diagram of methodology in this study.

## 3. Results

Among 153 patients enrolled in the studies 113 were malignant lesions. Among 113 malignant lesions 32 had high grading (G3) and 66 had HER2+ receptor.

**Figure 2:**
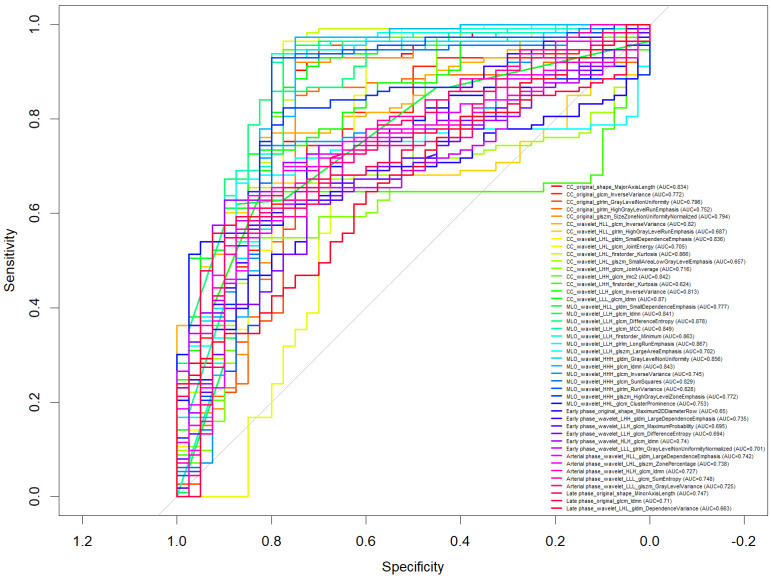
ROC curve of selected features to discriminate benign versus malignant lesions.

As shown in Figure 2, several features demonstrated strong predictive capability, with AUC exceeding 0.80 to differentiate benign from malignant lesions. Among the most discriminative features were:

- CC_wavelet_LLH_glrlm_LongRunEmphasis (AUC = 0.867)
- MLO_wavelet_LLH_glrlm_LongRunEmphasis (AUC = 0.867)
- CC_wavelet_LLH_firstorder_Kurtosis (AUC = 0.866)
- MLO_wavelet_LLH_glcm_Idmn (AUC = 0.841)
- MLO_wavelet_LLH_glrlm_RunVariance (AUC = 0.828)

Additionally, multiple features derived from wavelet-filtered images, especially in the LLH and HHH decompositions, showed consistently high performance, indicating the relevance of texture heterogeneity in both CC and MLO projections for lesion characterization.

Features extracted from DCE-MRI phases (early, arterial, delayed) also yielded informative predictors, such as Arterial phase_wavelet_LHL_glcm_Idmn (AUC = 0.748).

All five machine learning models demonstrated good performance (Table 3) in distinguishing benign from malignant breast lesions, with accuracy values above 0.80. Among the tested classifiers, the GBMachieved the highest overall performance, with an accuracy of 0.911, sensitivity of 0.970, and AUC of 0.907, indicating excellent discriminative ability and high true positive rate. The Random RF and NN models both achieved an accuracy of 0.844, with RF reaching an AUC of 0.905 and NN slightly lower at 0.861.

The LASSO also performed well, with an accuracy of 0.867, high sensitivity (0.909), and a balanced specificity of 0.750. Its AUC of 0.808 supports its reliability in identifying malignant lesions, although slightly lower than RF and GBM. The CART model, while still acceptable, had the lowest specificity (0.667) and AUC (0.798) among the five, suggesting a slightly reduced ability to correctly classify benign cases.

Overall, the GBM and RF models outperformed others in terms of AUC, confirming their robustness and effectiveness in radiomics-based classification of breast lesions.

**Figure 3:**
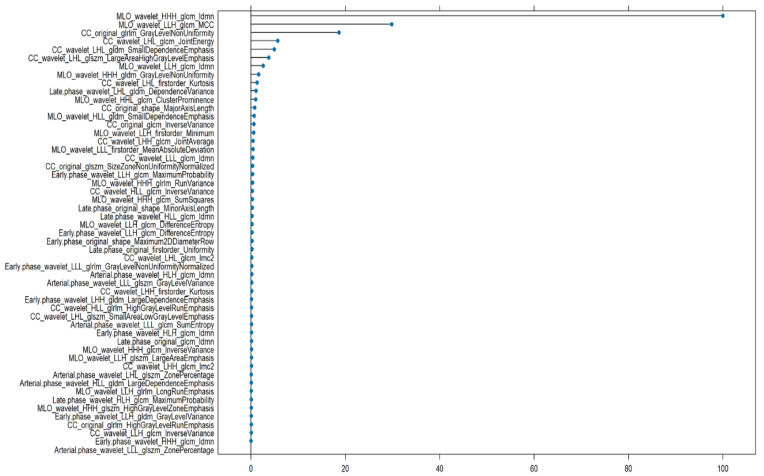
Radiomic features importance of GBM to discriminate benign versus malignant lesions.

**Figure 4:**
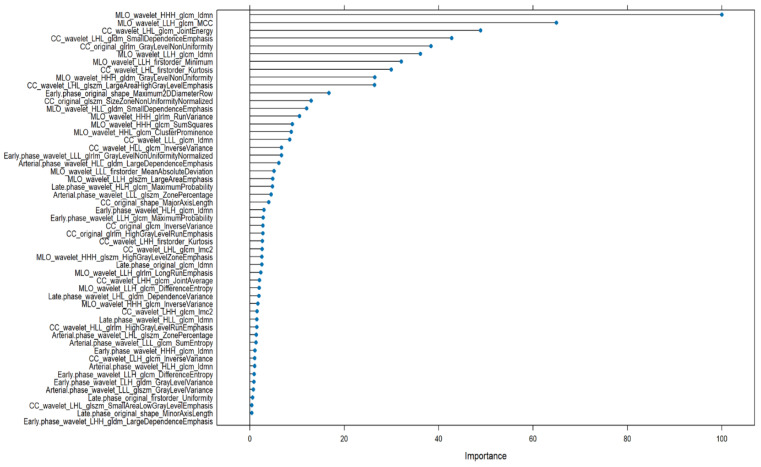
Radiomic features importance of RF to discriminate benign versus malignant lesions.

The top 10 most important features from GBM and RF models were compared and as shown in Figure 3 and Figure 4, a total of five radiomic features were found to be consistently ranked among the most relevant in both models:

- MLO_wavelet_HHH_glcm_Idmn
- CC_wavelet_LLH_glrlm_LongRunEmphasis
- CC_wavelet_LLH_glcm_Correlation
- CC_wavelet_LLH_glcm_Imc1
- CC_wavelet_LLH_glcm_Imc2

All five features are derived from wavelet-transformed images and computed from texture matrices such as the GLCMand the GLRLM. This highlights the central role of wavelet-based texture analysis in radiomics, particularly when characterizing lesion heterogeneity in mammographic views (CC and MLO).

The feature MLO_wavelet_HHH_glcm_Idmn, which measures local homogeneity, was the most influential in both models, suggesting that subtle local textural patterns within lesions are critical in distinguishing between benign and malignant cases.

**Figure 5:**
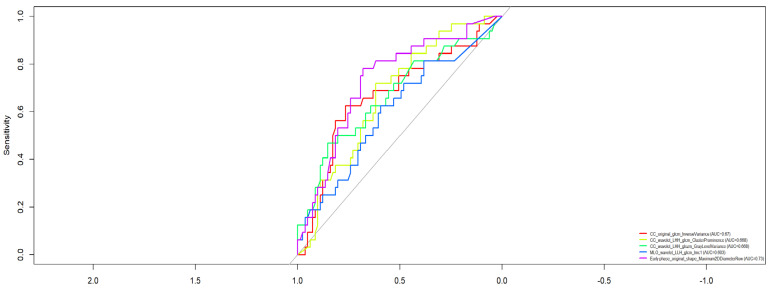
ROC curve of each feature selected by Elastic Net in the discrimination of low versus high grading malignant lesions.

Figure 5 presents the ROC curves of the most discriminative individual radiomic features for classifying low-grade (G1 + G2) versus high-grade (G3) malignant breast tumors. Overall, the performance of single features was moderate, with AUC values ranging from 0.603 to 0.73.

The most informative feature was Early phase_original_shape_Maximum2DDiameterRow, which reached an AUC of 0.73, indicating fair discriminatory capacity based on early-phase morphological shape characteristics.

Other relevant features included:

- CC_wavelet_LHH_glcm_ClusterProminence (AUC = 0.688)
- CC_wavelet_LHH_glszm_GrayLevelVariance (AUC = 0.688)
- CC_original_glcm_InverseVariance (AUC = 0.670)
- MLO_wavelet_LLH_glcm_Imc1 (AUC = 0.633)

These features derive from GLCM and GLSZM texture matrices extracted from both original and wavelet-transformed mammographic images, particularly from CC and MLO views.

Table 4 summarizes the performance of 5 classification models in distinguishing between low-grade (G1 + G2) and high-grade (G3) breast cancer lesions. Among the tested algorithms, the NN model showed the highest overall accuracy (0.848) with a balanced sensitivity (0.556) and specificity (0.958). This indicates its potential ability to identify high-grade tumors while maintaining strong detection of low-grade cases.

The LASSO regression model also demonstrated good classification performance, achieving an accuracy of 0.818, sensitivity of 0.667, and specificity of 0.875, making it the model with the highest true positive rate for G3 lesions. This suggests that LASSO may be particularly effective in detecting aggressive tumor phenotypes.

The RF and GBM models both reached high specificity (1.000) and perfect positive predictive value (1.000), but with limited sensitivity (0.333), indicating that they tended to classify more lesions as low-grade and may underperform in recognizing high-grade tumors.

The CART model was the least effective, with the lowest accuracy (0.758) and sensitivity (0.222), despite high specificity.

In terms of discriminative ability measured by AUC, RF (0.887) and GBM (0.843) outperformed other methods, followed by LASSO and NN (both 0.741). These results highlight a trade-off between sensitivity and specificity across models, where LASSO and NN offer more balanced performance for grading prediction.

**Figure 6:**
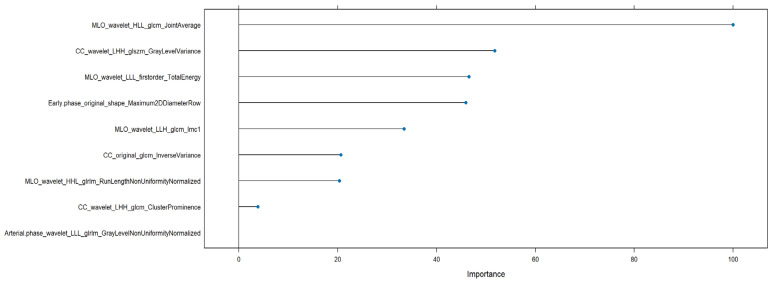
Radiomic features importance of NN to discriminate low versus high grade malignant lesions.

Figure 6 illustrates the variable importance scores derived from the NN model trained to discriminate between low- and high-grade malignant breast lesions. The most influential feature was MLO_wavelet_HLL_glcm_JointAverage, indicating a dominant role in the model’s decision-making. Other highly ranked features included CC_wavelet_LHH_glszm_GrayLevelVariance and MLO_wavelet_LLL_firstorder_TotalEnergy, which suggest that both spatial texture variability and signal energy distribution contribute significantly to grading differentiation.

Additional relevant features comprised Early.phase_original_shape_Maximum2DDiameterRow and MLO_wavelet_LLH_glcm_Imc1, reflecting the role of shape-related and information correlation metrics. Features like CC_original_glcm_InverseVariance and MLO_wavelet_HHL_glrlm_RunLengthNonUniformityNormalized provided complementary structural and intensity distribution information.

Lower in the ranking but still contributing were CC_wavelet_LHH_glcm_ClusterProminence and Arterial.phase_wavelet_LLL_glrlm_GrayLevelNonUniformityNormalized, indicating that both arterial phase and high-frequency wavelet features hold some discriminative power.

**Figure 7:**
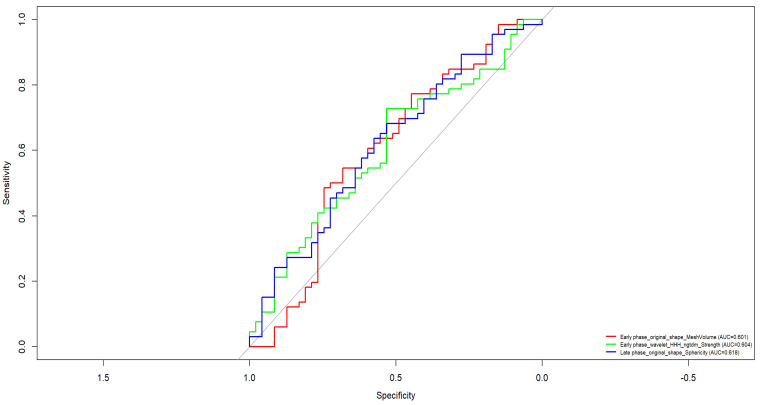
ROC curve of each feature selected by Elastic Net in the discrimination of HER2- versus HER2+ malignant lesions.

Figure 7 presents the ROC curve analysis for three selected radiomic features used to discriminate HER2-positive (HER2+) breast tumors. The best-performing feature was Late_phase_original_shape_Sphericity, which achieved an AUC of 0.618, suggesting a modest ability to differentiate HER2+ status based on the geometric regularity of the lesion.

Other relevant features included Early_phase_wavelet_HHH_ngtdm_Strength (AUC = 0.604), capturing the structural strength of high-frequency components in the early contrast-enhanced phase, and Early_phase_original_shape_MeshVolume (AUC = 0.601), a shape-based metric reflecting tumor size.

In the classification of HER2-positive versus HER2-negative lesions, model performance (Table 5) showed notable variability. The NN model achieved the best overall performance, with the highest accuracy (0.727), sensitivity (0.842), and balanced values of PPV and NPV (0.727 each), along with a fair area under the ROC curve (AUC = 0.669), indicating a reasonably good capability to identify HER2+ cases. The LASSO model demonstrated a high sensitivity (0.737) but lower specificity (0.286), leading to a modest overall accuracy (0.545) and AUC (0.530). The CART model reached perfect sensitivity (1.0), correctly identifying all HER2+ cases, but with a very low specificity (0.143) and a moderate AUC of 0.571, reflecting poor discriminative balance. The Random Forest (RF) and Gradient Boosting Machine (GBM) models showed perfect specificity (1.0) but very low sensitivity (0.105 and 0.0, respectively), with RF achieving a relatively higher AUC (0.701) despite its limited ability to detect positive cases.

**Figure 8:**
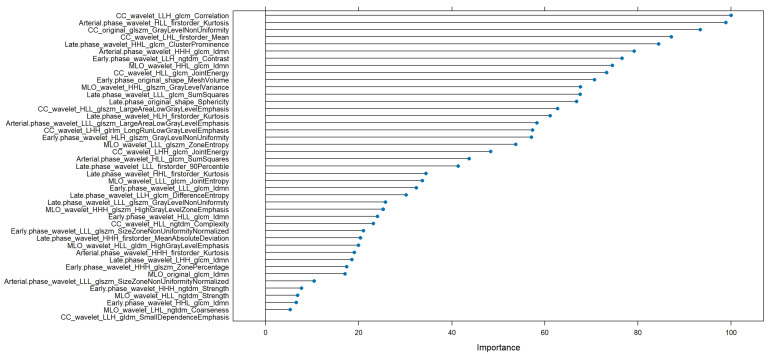
Radiomic features importance of NN to discriminate HER2+ malignant lesions.

The feature importance analysis for the NN model (Figure 8) in the HER2+ versus HER2− classification task revealed that a broad spectrum of radiomic features contributed to model performance. The most influential feature was CC_wavelet_LLH_glcm_Correlation, followed closely by Arterial.phase_wavelet_HLL_firstorder_Kurtosis and CC_original_glszm_GrayLevelNonUniformity. These features reflect critical aspects of texture and intensity distribution across both mammographic and MRI-derived wavelet-filtered images. Additional high-impact variables included CC_wavelet_LLH_firstorder_Mean, Late.phase_wavelet_HHL_glcm_ClusterProminence, and Arterial.phase_HHH_glcm_Idmn, highlighting the relevance of structural heterogeneity and signal uniformity. Several features from different imaging phases (early, late, arterial) and sequences (CC, MLO) appeared among the top ranks, underscoring the complementary nature of temporal and spatial information. The diverse contribution of features from various texture matrices (GLCM, GLSZM, NGTDM) further confirms the multifaceted radiomic signature distinguishing HER2 expression, although the overall discriminative power remained moderate.

## 4. Discussions

This study investigated the potential of radiomics combined with machine learning models applied to CEM and DCE-MRI to discriminate benign from malignant breast lesions and predict histopathological characteristics, including tumor grading and HER2 status. Our results demonstrate that radiomic features derived from both mammographic and MRI data provide valuable, non-invasive biomarkers for breast cancer characterization.

Among the 153 patients analyzed, 113 had malignant lesions, of which 32 were classified as high-grade (G3) and 66 were HER2-positive. The univariate analysis revealed that several individual radiomic features achieved excellent discriminatory performance in identifying malignancy, with AUC values exceeding 0.80. Specifically, features such as CC_wavelet_LLH_glrlm_LongRunEmphasis and MLO_wavelet_LLH_glrlm_LongRunEmphasis both achieved an AUC of 0.867. Additional discriminative features included CC_wavelet_LLH_firstorder_Kurtosis (AUC = 0.866) and MLO_wavelet_LLH_glcm_Idmn (AUC = 0.841). These features, derived from wavelet-transformed images and texture matrices, highlight the importance of tumor heterogeneity and spatial complexity in distinguishing malignancies.

All machine learning models performed well in the benign vs. malignant classification task. GBM achieved the highest accuracy (0.911) and AUC (0.907), followed closely by RF (AUC = 0.905) and LASSO (AUC = 0.808). NN and CART also showed strong performance. Notably, five features consistently ranked among the most important in both GBM and RF, MLO_wavelet_HHH_glcm_Idmn, CC_wavelet_LLH_glrlm_LongRunEmphasis, CC_wavelet_LLH_glcm_Correlation, CC_wavelet_LLH_glcm_Imc1, and CC_wavelet_LLH_glcm_Imc2, emphasizing the robustness of these textural descriptors across models.

In the grading task (G1 + G2 vs. G3), individual features performed moderately (AUC range: 0.603–0.73), with Early phase_original_shape_Maximum2DDiameterRow achieving the highest AUC. Multivariate analysis showed improved performance, with the NN model attaining the highest accuracy (0.848), supported by balanced sensitivity (0.556) and specificity (0.958). LASSO achieved the highest sensitivity (0.667), while RF and GBM maintained high specificity but low sensitivity. These findings suggest that while ensemble methods are robust in identifying low-grade lesions, models like LASSO and NN offer more balanced detection of high-grade tumors.

For HER2+ classification, the NN model again outperformed others, with the highest accuracy (0.727) and sensitivity (0.842), confirming its suitability for this molecular prediction task. LASSO also showed high sensitivity (0.737), while RF and GBM had perfect specificity but extremely low sensitivity. CART achieved 100% sensitivity but at the cost of poor specificity, indicating limited utility in clinical differentiation. Overall, AUC values were modest, with RF (0.701) and NN (0.669) leading.

Feature importance analysis for the NN model across both grading and HER2+ tasks highlighted a wide range of contributing radiomic signatures. For grading, the top features included MLO_wavelet_HLL_glcm_JointAverage, CC_wavelet_LHH_glszm_GrayLevelVariance, and Early.phase_original_shape_Maximum2DDiameterRow. For HER2+ prediction, CC_wavelet_LLH_glcm_Correlation, Arterial.phase_wavelet_HLL_firstorder_Kurtosis, and CC_original_glszm_GrayLevelNonUniformity were among the most relevant. These findings support the hypothesis that combining multiparametric radiomic descriptors from different views and imaging phases enriches the discriminatory capacity.

Compared to the strong performance observed for benign vs. malignant classification, the task of HER2+ prediction yielded lower AUC values across models, with the highest performance achieved by the neural network (AUC = 0.669). This modest discriminative ability highlights the increased complexity of molecular subtype classification using imaging data alone. Several factors may contribute to this limitation. First, HER2 overexpression represents a molecular phenotype that may not consistently translate into distinct macroscopic or radiomic imaging features detectable on CEM or DCE-MRI. Unlike malignancy, which often manifests with clear morphological or enhancement differences, HER2+ tumors may share overlapping imaging characteristics with HER2− subtypes. Additionally, while certain radiomic features, particularly from high-frequency wavelet transformations, were found to be informative, they may still lack the specificity required for robust HER2 prediction. It is also possible that the current feature set does not fully capture imaging correlations of HER2 biology, and future work may explore multi-omic integration, advanced deep learning, or dedicated imaging biomarkers (e.g., radiogenomic MRI signatures) to improve subtype classification.

For the benign vs. malignant classification, texture-based features derived from GLCM (Gray Level Co-occurrence Matrix) and GLRLM (Gray Level Run Length Matrix) were predominant. Notably, CC_wavelet_LLH_glrlm_LongRunEmphasis, MLO_wavelet_HHH_glcm_Idmn, and CC_wavelet_LLH_glcm_Correlation were consistently ranked among the top features in both GBM and RF models. These features reflect lesion heterogeneity, signal continuity, and spatial dependence—hallmarks of malignancy on imaging.

For tumor grading, features such as MLO_wavelet_HLL_glcm_JointAverage and CC_wavelet_LHH_glszm_GrayLevelVariance were repeatedly selected by NN and LASSO models, highlighting associations with internal textural uniformity and size zone variance, which may reflect tumor aggressiveness.

In the HER2+ classification task, top features included CC_wavelet_LLH_glcm_Correlation and Arterial.phase_wavelet_HLL_firstorder_Kurtosis, representing inter-pixel correlation and intensity dispersion within contrast-enhanced regions—suggesting that subtle enhancement patterns may reflect HER2 overexpression.

Across all tasks, wavelet-transformed features from both CC and MLO views were frequently selected, emphasizing the importance of multi-scale spatial analysis in capturing diagnostic and molecular information.

The comparison of the most important features was conducted post hoc, purely for interpretability purposes. It was aimed at identifying which radiomic features contributed most strongly to the predictive performance of each model. This step was not used for feature selection or model optimization and did not serve as a dimensionality reduction strategy. Given the relatively modest sample size, no significant computational advantage would have been gained. Instead, this analysis provided insights into the biological and structural relevance of key radiomic patterns.

In comparison with previous literature, our findings align with recent studies that demonstrated the potential of radiomics in predicting breast cancer molecular subtypes and histological grade [11,15,29,30,31,32,33,34,35,36,37,38,39,40,41,42,43,44,45]. For instance, La Forgia et al. [27] demonstrated that radiomic features extracted from CEM images were highly effective in distinguishing breast cancer molecular subtypes, particularly achieving strong classification performance for HER2-positive and triple-negative tumors. Their study reported an AUC exceeding 0.90 for HER2+ prediction, highlighting the discriminative power of enhancement-based textural and morphological descriptors. Similarly, Marino et al. [28] validated the potential of radiomics for identifying hormone receptor (HR) status, reporting robust model performance in classifying ER-positive and PR-positive tumors, both from CEM and MRI data, suggesting a translational application across imaging platforms.

The study by Stefano et al. [29] presents a comparative evaluation between machine learning-based radiomics and deep learning approaches for the classification of breast lesions in mammography. The authors extracted handcrafted radiomic features—such as shape, intensity, and texture descriptors—and compared their performance with that of a pre-trained convolutional neural network. While the deep learning model achieved strong performance in an end-to-end manner, the radiomics-based models demonstrated comparable classification accuracy, with the added advantage of greater interpretability. The study highlights that radiomics, when properly optimized and combined with machine learning algorithms, remains a reliable and transparent alternative to deep learning, especially in clinical decision-making contexts.

Furthermore, Leithner et al. [30] investigated multiparametric MRI-based radiomics to classify breast cancer into luminal, HER2-enriched, and triple-negative subtypes. Their models achieved AUC values ranging from 0.80 to 0.90, depending on the subtype, confirming the feasibility of non-invasive radiogenomic stratification. In parallel, Huang et al. [46] applied similar techniques and reported comparable accuracy, reinforcing the hypothesis that imaging phenotypes derived from radiomics closely reflect underlying molecular profiles. These studies support the findings of our work and emphasize the growing role of quantitative imaging biomarkers in breast cancer precision diagnostics.

Recent studies further reinforce the clinical potential of radiomics in oncology and in particular in breast cancer characterization and provide meaningful comparisons with our results [47,48,49,50,51,52]. Qi et al. [48] reviewed advances in breast cancer radiomics, emphasizing the growing use of multi-modal data and the critical need for external validation and standardized pipelines, challenges directly addressed by our multicenter design and robust cross-validation strategy. Yang et al. [49] focused on predicting Ki-67 expression using DCE-MRI radiomics, reporting predictive performance aligned with ours for histological grading, thus supporting the biological relevance of texture features in stratifying tumor aggressiveness. Additionally, Yang et al. [50] proposed a multi-modal integration of mammography and DCE-MRI to predict molecular subtypes, reporting improved classification of HER2+ tumors when combining modalities. These findings closely parallel our results, confirming that joint radiomics from CEM and DCE-MRI enhances the detection of aggressive phenotypes. Collectively, these recent contributions support the rationale, methodology, and outcomes of our study while also highlighting the need for future work on larger, prospective cohorts.

Nevertheless, some limitations must be acknowledged. Manual segmentation is labor-intensive and subject to operator variability. Although mitigated by consensus from experienced radiologists, future work should investigate semi-automatic methods to improve reproducibility. In particular, while the total cohort included 153 patients, subgroups used for molecular classification were relatively small. Specifically, only 32 lesions were classified as high-grade (G3) and 66 were HER2-positive. This class imbalance could potentially impact the generalizability and robustness of the trained models. To mitigate this, we applied the ROSE technique, a widely accepted method to synthetically balance datasets. However, synthetic oversampling cannot fully replace the diversity of real-world data and may introduce bias by overfitting to minority patterns. Therefore, we caution that the high sensitivity observed for HER2+ classification, particularly in models such as NN and CART, should be interpreted with care. Future studies involving larger, independent external validation cohorts are essential to confirm the reproducibility and clinical applicability of our models, especially for tasks involving minority class prediction such as HER2+ and high-grade tumors.

While convolutional neural networks (CNNs) have shown remarkable performance in end-to-end medical image classification, particularly in tasks involving spatial pattern recognition, we opted not to implement CNN-based architectures in the present study for several reasons. First, our methodological approach was grounded in handcrafted radiomics, which allows for explicit extraction of interpretable quantitative features from both contrast-enhanced mammography and DCE-MRI. This aligns with our primary goal of comparing classical machine learning algorithms on structured radiomic data across multiple classification tasks. Furthermore, CNNs typically require large, annotated datasets to achieve stable generalization and avoid overfitting. Given the moderate sample size of our study, especially in the G3 and HER2+ subgroups, applying CNNs would have posed a substantial risk of poor model generalizability without extensive data augmentation and external validation cohorts, which were beyond the scope of the current analysis.

Additionally, one of the strengths of radiomics lies in its interpretability: models such as GBM and LASSO enable feature importance ranking and biological insight into which image characteristics contribute to predictions. This level of transparency is difficult to achieve with deep learning models, which often function as “black boxes.” As such, for a multi-institutional study seeking both performance and clinical interpretability, the use of conventional machine learning on handcrafted features remains justified. Nevertheless, future work may involve comparative evaluations between radiomics-based models and deep learning architectures, either in isolation or as hybrid models, to explore potential gains in diagnostic accuracy and automated feature extraction.

## 5. Conclusions

This multicenter study demonstrated the promising role of radiomic analysis from combined CEM) and DCE-MRI in breast cancer characterization. Machine and deep learning models, trained on carefully selected features, showed excellent accuracy in differentiating benign from malignant lesions, with the Gradient Boosting Machine achieving an AUC of 0.907. For histological grading and HER2 status prediction, model performance was more modest but still informative, with neural networks achieving the best results in both tasks.

These findings confirm that radiomics-based models can support non-invasive tumor phenotyping by capturing subtle imaging patterns linked to underlying biological traits. Notably, the ability to distinguish high-grade tumors and HER2-positive subtypes, while less accurate than malignancy classification, suggests a direction for further optimization, such as integrating advanced features or multi-omics data.

Overall, this study contributes to the expanding field of imaging biomarkers, validating the utility of radiomic signatures across multiple imaging modalities. The results support future efforts to incorporate radiomics into clinical workflows for early diagnosis, risk stratification, and treatment planning in breast cancer care.

## Figures and Tables

**Table 1:** Magnetic resonance imaging scan settings.

Settings	T1-Weigthed DCE	Units
TR/TE/FA	4.4–5.1/2.0–2.4/15	ms/msdeg/
FOV	250–500 × 450–500	mm^2^
Matrix size	168–384 × 300–384	pixel
Slice thickness	3	mm
Intersection gap	0	mm
Pixel spacing	0.89–1 × 0.89–1	mm^2^

**Table 2:** Hyperparameter tuning for each model.

Model	Tuned Hyperparameters	Search Strategy	Selection Criterion
Elastic Net Logistic Regression	α = 0.5 (Elastic Net) λ ∈ [0.001, 0.1] (100 values)	Cross-validation via cv.glmnet()	Minimum cross-validated error (lambda.min)
Random Forest	mtry = √(number of features) ntree = 200 min.node.size = {5, 10, 20}	Grid search	Highest AUC
Gradient Boosting Machine	n.trees = {50, 100, 200} interaction.depth = {2, 4, 6, 8} shrinkage = 0.01 n.minobsinnode = 10	Grid search	Highest AUC
Neural Network	size = {2, 4, 6} decay = {0.1, 0.5}	Grid search	Highest Accuracy
Decision Tree (CART)	cp selected via pruning minsplit = 15 maxdepth = 15	Cross-validation with cost-complexity pruning	Lowest cross-validated error

**Table 3:** Model performance metrics.

Model	Accuracy	Sensitivity	Specificity	PPV	NPV	AUC
RF	0.844	0.879	0.75	0.906	0.692	0.905
LASSO	0.867	0.909	0.75	0.909	0.75	0.808
GBM	0.911	0.97	0.75	0.914	0.9	0.907
NN	0.844	0.879	0.75	0.906	0.692	0.861
CART	0.822	0.879	0.667	0.879	0.667	0.798

**Table 4:** Model Performance—G1 + G2 vs. G3 Classification.

Model	Accuracy	Sensitivity	Specificity	PPV	NPV	AUC
RF	0.818	0.333	1.0	1.0	0.8	0.887
LASSO	0.818	0.667	0.875	0.667	0.875	0.741
GBM	0.818	0.333	1.0	1.0	0.8	0.843
NN	0.848	0.556	0.958	0.833	0.852	0.741
CART	0.758	0.222	0.958	0.667	0.767	0.734

**Table 5:** Model Performance for HER2+ Classification.

Model	Accuracy	Sensitivity	Specificity	PPV	NPV	AUC
RF	0.485	0.105	1.0	1.0	0.452	0.701
LASSO	0.545	0.737	0.286	0.583	0.444	0.53
GBM	0.424	0.0	1.0	NA	0.424	0.658
NN	0.727	0.842	0.571	0.727	0.727	0.669
CART	0.636	1.0	0.143	0.613	1.0	0.571

## Data Availability

Data are reported in the manuscript and at link https://zenodo.org/records/16993747.
